# Spelling Intervention in Post-Stroke Aphasia and Primary Progressive Aphasia

**DOI:** 10.3233/BEN-2012-110240

**Published:** 2013

**Authors:** Kyrana Tsapkini, Argye E. Hillis

**Affiliations:** ^1^Departments of NeurologyJohns Hopkins MedicineBaltimoreMDUSA; ^2^Physical Medicine and RehabilitationJohns Hopkins MedicineBaltimoreMDUSA; ^3^Department of Cognitive ScienceJohns Hopkins UniversityBaltimoreMDUSA

**Keywords:** Spelling, intervention, PPA, stroke, post-stroke aphasia

## Abstract

Spelling–a core language skill–is commonly affected in neurological diseases such as stroke and Primary Progressive Aphasia (PPA). We present two case studies of the same spelling therapy (learning of phoneme-to-grapheme correspondences with help from key words) in two participants: one who had a stroke and one with PPA (logopenic variant). Our study highlights similarities and differences in the time course of each indivdual's therapy. The study evaluates the effectiveness and generalization of treatment in each case, i.e. whether the treatment affected the trained items and/or untrained items, and whether or not the treatment gains were maintained after the end of therapy. Both participants were able to learn associations between phonemes and graphemes as well as between phonemes and words. Reliable generalization to untrained words was shown only for the participant with post-stroke aphasia, but we were not able to test generalization to untrained words in the individual with PPA. The same spelling therapy followed a different time course in each case. The participant with post-stroke aphasia showed a lasting effect of improved spelling, but we were unable to assess maintenance of improvement in the participant with PPA. We discuss these differences in light of the underlying nature of each disease.

## 1. Introduction

Primary Progressive Aphasia (PPA) is a neurodegenerative disease characterized by language impairment at its onset. Although other cognitive functions are affected in the course of PPA, language deficits remain the most characteristic and frequent symptom. Furthermore, it has been shown that spelling is one of the earliest affected language skills [1]. There is currently no available pharmacological treatment for these patients; however, a few studies document behavioral interventions, mostly of naming [2-5,5-12) and only one of spelling [13]. Several variants of PPA have recently been identified: logopenic, semantic, and nonfluent/agrammatic [14]. These are associated with distinct brain pathologies and different regions of brain atrophy. Different variants of PPA show atrophy in brain areas associated with performance in distinct language tasks such as grammatical processing, semantic processing, and fluency [15,16]. However, the unique features (language characteristics and areas of atrophy) that differentiate PPA variants at early-to-middle stages tend to lose their distinctiveness later on [16]. Many patients become nonverbal, but may retain some ability to write and type. Because they may depend on writing to communicate, recognizable spelling is crucial for basic communication. As for all people in today's society, use of e-mail, computers, and other texting that depends on spelling is crucial for basic activities. Spelling is not only one of the first language functions affected in all three variants of PPA but also has prognostic value for the evolution of the disease. Different variants of PPA have distinct spelling deficits. A recent study from our laboratory has identified early spelling patterns that predict the variant of PPA into which the patient is like-

ISSN 0953-4180/13/$27.50 © 2013 – IOS Press and the authors. All rights reserved ly to evolve, when only naming and spelling are affected (such that the patient does not yet meet criteria for any one variant). Our study further determined which cognitive processes underlying spelling are affected in each of the PPA variants [1,17].

In brief, there are three main cognitive processes involved in spelling of familiar words to dictation: recognition of the spoken word (access to the stored phonological representation of the word), access to the meaning of the word (access to the lexical-semantic representation of the word) and access to the correct spelling of the word (the stored orthographic lexical representation). In the case of unfamiliar words or pseudowords (non-existing, word-like utterances), the mechanism recruited is a sublexical letter-to-sound conversion (phoneme-to-grapheme correspondence mechanism). Spelling of all words and nonwords requires temporary storage of the sequence of letters in working memory–a storage system called the 'graphemic buffer' – while the individual letters are being written or spelled out loud. All of these mechanisms have been described in a series of case studies mainly of stroke patients [18–26].

In stroke and PPA any of these mechanisms (as well as combinations of them) may be disrupted, and therapeutic interventions have been shown to improve patients' communication. One of the most affected spelling processes in stroke is the phoneme-to-grapheme (PG) conversion mechanism: the mechanism that applies letter-sound associations during spelling and provides a way to write unfamiliar words. Successful remediation for this mechanism as described in relevant cognitive neuropsychological models has been shown to have beneficial effects for patients post-stroke [27–29]. Few studies treat spelling disorders and in particular treat the PG mechanism. Possible reasons include; (a) most literate subjects write lexically by directly accessing the orthographic lexicon, and (b) the PG mechanism is more useful in languages with transparent orthographies – one-to-one PG correspondences, like Italian but not English. Luzzatti et al. [29] include a comprehensive list of all studies treating the sublexical level [27,30–32] in spelling, but the results lack homogeneity, ranging from no effects to improvement involving either treated-only or both treated and untreated items. We review three studies using treatment of the PG conversion mechanism with generalizable and sustainable results [27–29]. Hillis [27] first used this approach to remediate spelling in a stroke patient who was not able to spell unfamiliar words or syllables to dictation. The patient was mute and was taught PG conversion in order to use to text-to-speech synthesizer. She learned correspondence between phonemes and graphemes using a cuing hierarchy involving key words and maintained this system 12 months after the end of treatment. She could successfully spell monosyllabic words, but for longer words or sentences she relied on her impaired system of orthographic access resulting in frequent semantic errors. Luzzatti et al. [29] described the rehabilitation of dysgraphia in two Italian patients after stroke. Emphasis was given to phonological analysis of the auditory string to be written. After treatment, patients could spell at normal levels on most subsets of therapy items as well as in spontaneous writing and written naming tasks. Therapy gains were maintained 6-18 months post-treatment. Greenwald [28] documented treatment of severe global agraphia after stroke. Spelling performance on both regular and irregular words improved after treatment of sublexical spelling (PG conversion rules); however, regular words improved more than irregular words. With treatment, the patient's global agraphia evolved to surface dysgraphia but follow-up results were not mentioned.

There are few treatment studies in PPA [33] and these studies have focused on treatment of word retrieval with encouraging results for trained items, primarily in the semantic variant of PPA [4–7,11]. There are also a few studies of word retrieval treatment in non-fluent/agrammatic variant PPA [7–10], with no evidence of lasting effects. The logopenic variant of PPA (lvPPA) has received minimal attention. Only a few studies have documented treatment of language functions in lvPPA: two studies with naming interventions [2,3] and one with a spelling intervention [13]. In PPA, any of the aforementioned spelling mechanisms may be disrupted [1]. Phoneme-to-grapheme conversion (PGC), for example, has been shown to be disrupted mostly in patients with the logopenic variant PPA and nonfluent variant PPA, and spared in patients with semantic variant PPA. In general, there is a remarkable paucity of studies of spelling and even more so of treatment of spelling deficits in PPA.

We are aware of only one study of a spelling intervention in PPA. Rapp and Glucroft [13] documented a lexical (word-level) spelling intervention for a PPA patient (logopenic variant, although not mentioned explicitly), which examined the effectiveness of a non-intensive spelling therapy using a spell-study-spell procedure. They found that a spelling intervention can provide immediate and short-term benefits for dysgraphia in PPA, without, however, generalization to untrained items. Also, at 12 month follow-up, all word sets (trained, repeated, homework, and control) had declined even in comparison to pre-intervention baseline.

In our study we provided spelling therapy to an individual with logopenic variant PPA and compared results to results of the same therapy provided to an individual with post-stroke aphasia. Both participants experienced difficulties with spelling (dysgraphia) and in particular with PGC. Consistent with the severity of his initial aphasia, the one who had post-stroke aphasia had extensive left hemisphere damage. As far as we know, this is the first time such a therapy has been used in PPA. The aims of the present study were to: (1) report on the effectiveness of spelling therapy using letter-to-sound associations (PGC) in PPA; (2) directly compare the effectiveness of the spelling therapy used between PPA and severe damage from chronic stroke; (3) consider the similarities and differences in the course and pattern of spelling therapy results between the two types of dysgraphias (acquired, recovering vs. progressive) to evaluate possible compensatory mechanisms that could be recruited in each case.

## 2. Methods

### 2.1. Participants

WCR, was a 62-year-old right-handed man, with an MA in history who presented with a large, left-sided, middle-cerebral artery stroke 3 years before testing. The stroke affected most of left hemisphere, sparing only occipito-temporal and medial frontal areas but not sparing their connections to lateral frontal and parietal areas. Initial MRI revealed a large, left middle cerebral artery stroke and an incidental finding of a completely occluded right carotid artery. Initially, he was globally aphasic, but had improved mainly through rehabilitation and probably physiological reinstitution at least in the first year. Three years post-stroke, WCR lived independently, taking care of his own bills, and all of his own needs. His aphasia type was unclassifiable; he was classified by the Western Aphasia Battery (WAB) as anomic [34], but he had "asyntactic" auditory comprehension on sentence comprehension tasks. He was administered the WAB and supplemental tests of sentence comprehension and working memory (see Table 1). His scores in a sentence-picture matching task were at 85% correct for active sentences, 80% for passive, 65% for cleft subject (e.g., "It was the man that kicked the girl."), 55% for cleft object (e.g.,"It was the niece that the father kicked.), 48% for semantically reversible (e.g., "The dog chased the boy."), and 95% for irreversible sentences (e.g., "The girl kicked the ball."). In an enactment task (in which he had to enact the sentence with paper dolls), he was 100% correct with active sentences, 35% with passive, 95% with cleft subject, 50% with cleft object, 55% with reversible and 90% with irreversible sentences. Notable sub-scores on the WAB (see Table 1 for a summary of results) are: 90% correct in sentence repetition, 100% correct in object naming, 100% correct in sentence completion and 100% correct in responsive speech (naming to definition). His spontaneous speech was generally fluent and well articulated, but he had hesitations for word finding and frequent circumlocutions. He followed and participated in conversations without difficulty.

FSE, was a 62-year-old right-handed woman with a BA degree who was an elementary teacher but retired 10 years before testing. Six years before treatment she started having difficulties forming sentences and finding words. She was first diagnosed with logopenic variant PPA three years prior to therapy on the basis of history, neurological examination, imaging, and neurocognitive assessment completed at Johns Hopkins. When she first presented, her speech was fluent and well-articulated but with frequent hesitations for word finding and many circumlocutions. MRI showed left greater than right temporoparietal atrophy. During the course of the disease her speech became full of stereotypic phrases and hesitations for word finding. She had difficulty formulating and repeating sentences. She could repeat the partial meaning of sentences but not the exact words. She made phonemic and semantic paraphasias. Her performance on the WAB and other neuropsychological tests is summarized in Table 1.

#### 2.1.1. Spelling impairments of the two participants

WCR: The participant was tested before intervention on Johns Hopkins Dysgraphia Battery when he presented to the clinic, 3 years post-stroke. As shown in Table 2, his performance on words and pseudowords was very impaired (11% accurate on words and 0% on nonwords). The following information refers to his performance on words: Nouns were somewhat better preserved than verbs, but the difference was not significant (21% vs. 7% accurate). There was not a significant concreteness effect, although abstract words were slightly less accurate than concrete words (5% vs. 19% accuracy on abstract vs. concrete words). He did not show any frequency or regularity effect either (22% and 19% accurate on high vs. low frequency words and 33% vs. 36% on high vs. low regularity words).

Table 1
Performance of patients WCR and FSE in the Western Aphasia Battery (WAB) and other neuropsychological tests

| Tests from the WAB | WCR (8/14/2009) | FSE (6/10/2009) |
|---|---|---|
| Overall aphasia quotient: | 85 | 68 |
| Aphasia type: | anomic (but asyntactic) | conduction |
| Spontaneous speech total: | 15/20, 75% Correct | 15/20, 75% Correct |
| – Information content: | 10/10, 100% Correct | 10/10, 100% Correct |
| – Fluency, grammatical competence: | 5/10, 50% Correct | 5/10, 50% Correct |
| Auditory verbal comprehension | 86% correct | 88% correct |
| – Yes/no questions: | 54/60, 90% Correct | 51/60, 85% Correct |
| – Auditory word recognition: | 55/60, 92% correct | 60/60, 100% correct |
| – Sequential commands: | 60/80, 75% correct | 64/80, 80% correct |
| Repetition total: | 90/100, 90% correct | 56/100, 56% correct |
| Naming and word finding | | |
| – Object naming: | 60/60, 100% correct | 27/60, 45% correct |
| – Word fluency: | 8/20, 40th percentile | 3/20, 15th percentile |
| – Sentence completion: | 10/10, 100% correct | 8/10, 80% correct |
| Responsive speech: | 10/10, 100% correct | 8/10, 80% correct |
| *Trail Making Test* | Part A: 128 sec<br>Part B: not completed | Part A: 42,<br>Part B: 16 |
| *Digit Span* | Forward: 4<br>Backward: 3 | Forward: 3<br>Backward: 2 |
| *Word Span* | Forward: 3<br>Backward: 2 | Forward: 2<br>Backward: 2 |

He only showed a significant word-length effect (43% correct on 4-letter vs. 7% on 5-letter vs. 14% on 6-letter vs. 7% on 7-letter and 0% on 8-letter words correct). His errors were mostly phonologically implausible nonwords (e,g., child -> chies; strange -> staging; happen -> happist; brother -> browets). WCR had a pronounced length effect that characterizes patients with impairment at the level of graphemic buffer, a working memory system that temporarily holds information about identity and sequence of letters while the word and nonword is written. In combination with his 0% accuracy on nonwords, his pattern of performance was congruent with impairments both at the level of the graphemic buffer and at the level of sublexical mechanisms for phoneme-grapheme conversion. We decided to treat his sublexical route – the phoneme-grapheme conversion mechanism – as a first step, to give him some rules he could rely on to transcribe from the auditory to the written word. Treatment of the graphemic buffer impairment was planned at a later stage.

FSE: Before treatment, on the Johns Hopkins Dysgraphia Battery, FSE spelled words and pseudowords with approximately equal accuracy (65.5 vs. 61.8% accuracy). The following information refers to her performance on words: There was no effect of grammatical class (68% correct in nouns and verbs, 61% in adjectives and 65% in function words). She did, however, show a concreteness effect (90.5% correct for concrete and 52% for abstract words). There was also a significant frequency effect (88% correct for high frequency words and 72% for low frequency ones). There was no significant difference between high/low regularity words (80.0 vs. 83.8% correct). There was no significant effect of word length; she correctly spelled 85.7% of 4-letter vs. 92.9% of 8-letter words (ns different by chi square). Only 15.2% of her errors were phonologically plausible errors (rooster-> rouster; pigeon-> picheon). Most of her errors were phonologically similar word errors (bright-> bride, brick; chain-> chant) or mixed phonologically and semantically similar words including morphological errors (absence-> absent; speak-> speech; begin-> begun; jury-> juror). These types of errors have been proposed to occur as a result of an interaction between information from the semantic system and partial information from the impaired phoneme-to-grapheme conversion (PGC) mechanism, which each access lexical representations semantically or phonologically related to the target. According to this "summation hypothesis", lexical representations that are both semantically and phonologically related to the target will be activated nearly as much as the target is activated. When the target lexical orthographic representation is unavailable (due to damage at that level), the semantically and phonologically related representation might be activated for output instead [35]. Thus, we hypothesized that FSE was impaired both (1) in accessing orthographic lexical representations for output, and (2) in phoneme-to-grapheme conversion (PGC). We decided to treat the latter mechanism, as improvement might also improve access to orthographic lexical representations.

### 2.2. Treatment

For the spelling therapy, 30 English sounds were selected representing most common word-initial English phonemes (speech sounds) and were divided into 3 sets using a counterbalanced design with respect to the frequency of the initial phoneme. Then a set of 30 English words starting with these sounds were selected to be used as prompts to help the patients relate each sound to a grapheme (abstract letter identity or sequence of letter identities). The sequence of events in the therapy sessions was as follows: The participant was asked to write the letter or combination of letters corresponding to a particular phoneme, e.g., 'sh'. If the participant was correct, then s/he was reinforced, and asked to write a word that starts with this sound. However, if the participant could not write the correct letter, s/he was asked to think of a word that starts with this sound or, if needed, was provided with such a word (prompt); then s/he was asked to write the word and then instructed explicitly to associate that particular phoneme to the particular word and initial grapheme. Each session consisted of teaching the correct phoneme-grapheme correspondence of 10 initial word phonemes using 10 common English words. When the patient reached criterion for two consecutive sessions and a minimum of 3 sessions per set (80% correct, i.e., 8/10 of phoneme-grapheme or phoneme-word correspondences) for each set, s/he would continue to the second set and then to the third set. For each set, trial accuracy and response times for the letter and the word responses were recorded. The 3 sets were matched for several linguistic variables so that they were equivalent in difficulty. Baseline evaluation before each therapy session required that the participant would choose – from a series of possible initial letters or letter sequences of English (30 choices) written in front of him or her – the letter, or letter sequence, that corresponded to the phoneme the examiner provided each time. The same measurement was used as an independent evaluation of the therapeutic outcome after therapy. Participants received approximately one therapy session per week, with the duration of one to two hours each. Both liked to talk quite a bit during the session, accounting for the variability in the duration of the sessions.

### 2.3. Assessment

Follow-up assessment probed all three sets of trained phoneme-grapheme and phoneme-word correspondences to identify whether or not the participant had retained knowledge of the trained items. We also looked at generalization effects, that is, effects of training to untrained items. We trained all possible initial phoneme-grapheme correspondences of English in 3 sets (30 items), and compared trainedto untrained items directly. Differences in baseline measures in pre- and post-therapy accuracy for phoneme-grapheme correspondences for each participant were evaluated using chi-square or Fisher's exact tests. We also compared the improvement of each participant in trained items using a chi-square test to evaluate whether either of them improved more than the other. Additionally, we evaluated pre- and post-therapy accuracy for graphemes and for words for the trained items for each participant using Fisher's exact tests. Finally, we did follow-up assessments when possible (for WCR only).

## 3. Results

Both participants completed all three sets of 10 phoneme-grapheme correspondences that comprised the spelling therapy; that is, they were able to learn the new associations and benefit from the intervention. However, each of them showed a different pattern of performance in the course of training. First, they completed the training in different lengths of time. WCR, the one with post-stroke aphasia, completed the training in 25 sessions; FSE, the one with PPA completed the training in 11 sessions. Furthermore, they showed a different pattern in learning of phoneme-grapheme and phoneme-word associations. Results are presented below in detail.

1. We first addressed the question of whether the participants showed generalization of treatment by comparing the percentage of trained vs. untrained items before and after the first set of the therapeutic intervention. Given that there are only 30 initial phoneme-grapheme correspondences in English and we trained 10 of them in each set we looked for generalization to untrained items before and after the first set. We used chi-square or Fisher's exact test to evaluate whether there was significant improvement in phoneme-grapheme correspondence (see Figs 1a and 1b for results in each set). We also evaluated generalization of treatment to other

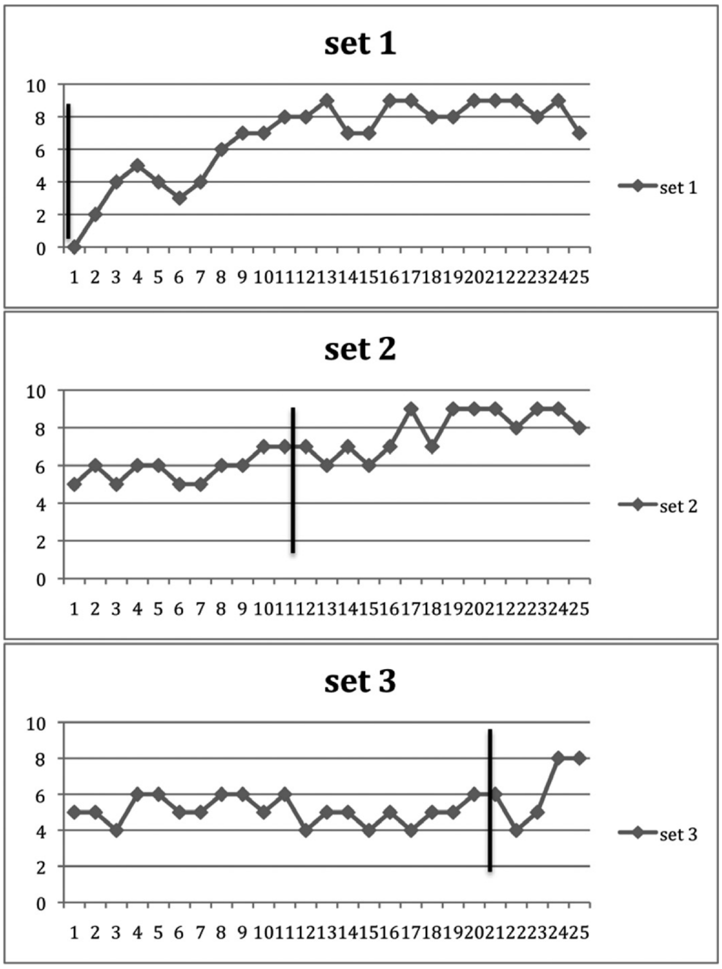

Fig. 1a. Effect of training in trained and untrained items of all 3 sets for patient FSE. Each dot corresponds to correct items per session for letter-phoneme correspondences. Horizontal axis corresponds to the number of therapy sessions and vertical axis corresponds to the level of success (out of 10 items per set). Vertical bars show when training began for each set.

spelling tasks before and after treatment (spelling to dictation, pointing to named letters) for WCR.

For FSE, we found that there was not a significant difference in the trained or untrained phoneme-grapheme correspondences between the beginning and the end of set 1 because she was not severely impaired at baseline: (Fisher's exact for trained: $p = 0.3$; Fisher's exact for untrained: $p = 1$). However, she improved with each set as it was trained (Fig. 1a). Also, there was an almost significant difference between the trained phoneme-word associations (Fisher's exact: $p = 0.057$, see Fig. 2). Therefore, in the first set she showed no significant improvement in trained items nor generalization to untrained items in phoneme-grapheme correspondences but she did show an improvement in her phoneme-word correspondences. Furthermore she reached criterion more quickly for the consecutive training sets indicating that there had been at least some overall generalization to untrained phonemes. Unfortunately it was not possible to test her thoroughly post-intervention in spelling of unpracticed words and nonwords – such as the ones included in the JHU Dysgraphia battery – in order to demonstrate functional generalization. Generalization to other untrained tasks such as pointing to named letters, was, however, demonstrated by a significant improvement in her performance before and after intervention (Fisher's exact $p = 0.021$).

For stroke patient WCR there was a significant difference in trained but not in untrained phoneme-grapheme correspondences after the first set (Fisher's exact for trained: $p = 0.0007$; Fisher's exact for untrained: $p = 0.65$). Therefore, in the first set he showed no generalization to untrained phonemes. There was also no

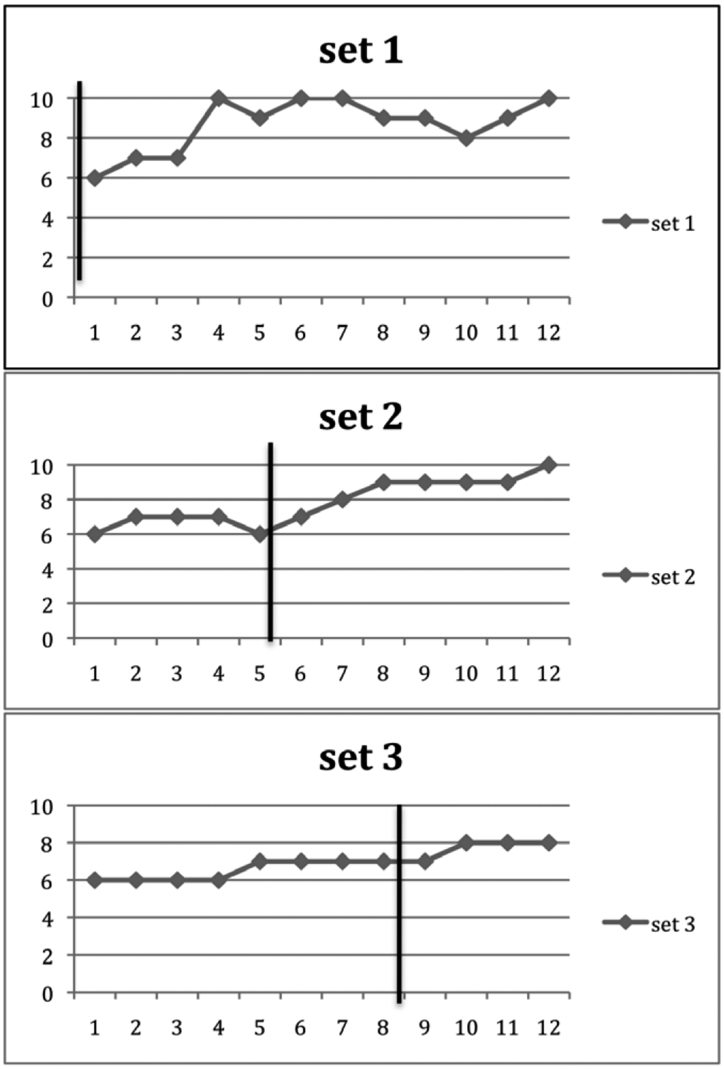

Fig. 1b. Effect of training in trained and untrained items of all 3 sets for patient WCR. Each dot corresponds to correct items per session for letter-phoneme correspondences. Horizontal axis corresponds to the number of therapy sessions and vertical axis corresponds to the level of success (out of 10 items per set). Vertical bars show when training began for each set.

difference between the trained phoneme-word associations before and after the first set (Fisher's exact: $p = 0.17$). WCR, like FSE, also reached criterion more quickly for each successive test suggesting that he generalized his new skill to untrained items (Figure 1b). However, generalization to untrained items and tasks was demonstrated by dramatically increased accuracy of spelling words to dictation in the JHU Dysgraphia battery (see Table 2) as well as pointing to named letters (chi-square (1, $N = 60$) = 7, $p < 0.001$). For example, WCR's performance in open class words improved significantly from 11 to 27% (chi-square (1, $N = 168$) = 6.5, $p < 0.01$). Pertinent to the therapy, his spelling of high probability words (words with regular phoneme-to-grapheme correspondence) improved significantly from 33 to 73% (chi-square (1, $N = 60$) = 8.1, $p < 0.001$); whereas his spelling of low probability (words with irregular phoneme-to-grapheme correspondence) improved, but not significantly (from 36 to 49%, chi-square (1, $N = 80$) = 2.1, $p < 0.1$, ns). His performance in nonwords included in the JHU battery did not improve significantly. A possible reason is that these nonwords are more than 4-letter, usually 2-syllable pronounceable nonwords that can be spelled with a minimum of 5–7 letters. This presented a further problem for WCR who also had a graphemic buffer impairment as manifested in the difference of his performance between 4-letter and longer words (see Table 2);

Table 2
WCR's performance in the JHU battery before and after spelling intervention

| JHU Dysgraphia Battery | Before treatment | After treatment |
|---|---|---|
| Part of speech | | |
| Open class words | 9/84, 11% | 23/84, 27% |
| Nouns | 6/28, 21% | 12/28, 43% |
| Verbs | 2/28, 7% | 5/28, 18% |
| Adjectives | 1/28, 4% | 6/28, 21% |
| Function words | 1/20, 5% | 1/20, 5% |
| Nonwords | 0/34, 0% | 1/34, 3% |
| Concreteness | | |
| Concrete words | 1/21, 5% | 8/21, 38% |
| Abstract words | 4/21, 19% | 5/21, 24% |
| Word length | | |
| 4-letter | 6/14, 43% | 10/14, 71% |
| 5-letter | 1/14, 7% | 3/14, 21% |
| 6-letter | 2/14, 14% | 4/14, 29% |
| 7-letter | 1/14, 7% | 1/14, 7% |
| 8-letter | 0/14, 0% | 0/14, 0% |
| Probability (PG regularity) | | |
| High probability | 10/30, 33% | 22/30, 73% |
| Low probability | 29/80, 36% | 39/80, 49% |
| Frequency | | |
| High frequency | 32/146, 22% | 65/146, 45% |
| Low frequency | 27/146, 19% | 46/146, 32% |

a difference maintained in the post-treatment evaluation as well. Overall, WCR showed clear indications of generalization of treatment to untrained items.

2. We also wanted to determine whether either of the patients improved more than the other. We compared the degree of improved performance (difference in correct phoneme-grapheme correspondences between the beginning and the end of therapy) for each patient to the utmost potential improvement for each patient: chi-square (1, $N = 51) = 0.003, p > 1$, showing that there was no difference in improvement between the two patients. Another indication that the patients were benefitting from therapy is that both of them reached criterion for the final set in fewer sessions than those of the first. Patient FSE completed the first set in 5 sessions, the second in 3 sessions and the third in 4 sessions. Patient WCR completed the first set in 11 sessions, the second in 9 sessions and the third in 5 sessions. (see Figs 2 and 3 for performance on letters and words in each set).

3. In order to evaluate whether either patient had improved more in any domain, that is, the phoneme-grapheme associations or phoneme-word associations, we also compared the difference between their initial and final performances in phoneme-grapheme and phoneme-word associations. WCR showed a dramatic improvement in phoneme-grapheme associations from 10/30 to 24/30 (chi-square (1, $N = 60) = 7, p < 0.001$) and a significant improvement in phoneme-word associations (Fisher's exact $p = 0.02$) (see Fig. 3). FSE did show a significant difference in phoneme-grapheme associations learned in therapy (Fisher's exact $p = 0.021$): her performance improved from 20/30 to 28/30 correct. She also showed an improvement in phoneme-word associations learned in therapy (chi-square (1, $N = 60) = 8.4$, $p < 0.01$). (see Fig. 3). These results indicate that both participants were helped in both domains.

4. We were able to perform a 6-month follow-up session only for WCR who had post-stroke aphasia. WCR showed good maintenance of learned phoneme-grapheme conversion (see Table 3). Performance was not significantly different from that at the end of training for any measurement; for phoneme-grapheme associations: chi-square (1, $N = 60) = 0$, $p > 1$; for phoneme-word associations (Fisher's exact $p = 0.33$). Unfortunately, at the time of the scheduled follow-up appointment FSE's condition in other cognitive domains (recognition memory, attention, executive functions) had deteriorated to the point she had to be moved to a nursing home facility for people with dementia and could not be tested.

## 4. Discussion

We documented two case studies of spelling therapy (training of the phoneme-to-grapheme conversion system) in post-stroke aphasia and PPA. We compared the course and outcomes of therapy in these two participants to determine whether treatment of phoneme-to-grapheme conversion previously applied successfully in post-stroke aphasia [27–29] can be applied in the case of neurodegenerative diseases such as PPA. Only one study in the literature [13] shows that individuals with PPA may benefit from a spelling intervention at the word-level without, however, generalization to unpracticed items. Our study validates alternative approaches. The therapy of these two participants showed similarities but also differences. Both participants learned all three training sets of phoneme-grapheme associations and reached criterion in all of them in fewer sessions, showing that even in a degenerative disease learning is possible when the therapy is targeted. There were indications of possible generalization of treatment to other items and tasks for the PPA participant and reliable generalization to untrained items and tasks for the participant with post-stroke aphasia as shown in spelling to dictation on the JHU Dysgraphia battery and pointing to named letters. The participant with PPA required fewer

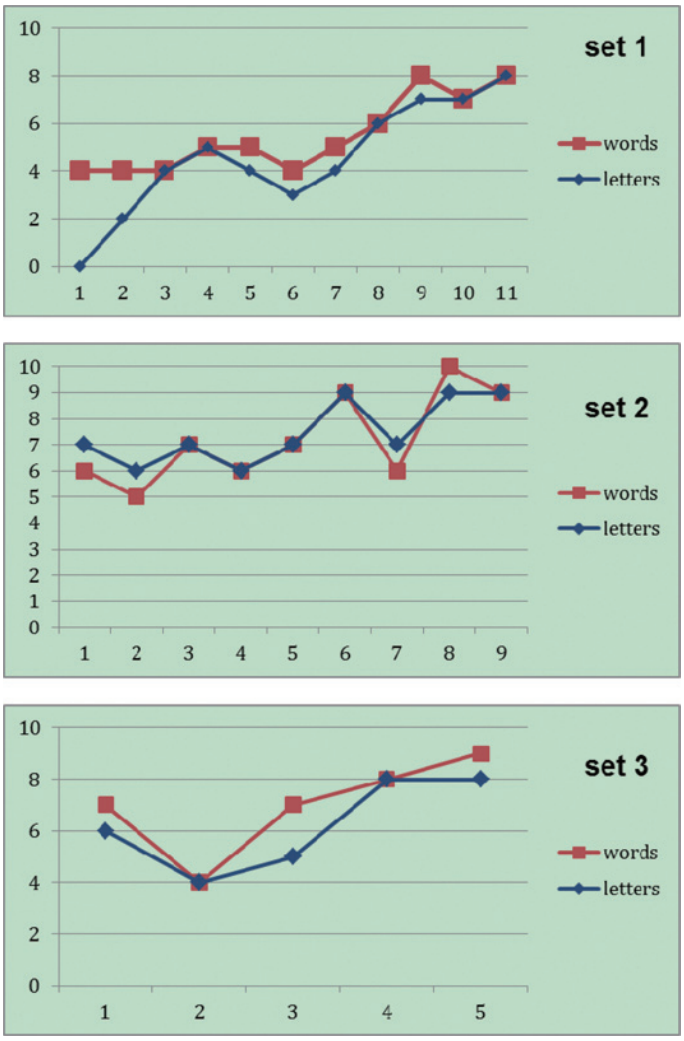

Fig. 2. Effect of training on trained items in each session for letter- and word-sound correspondences for patient FSE. Each dot corresponds to correct items per session for letter- and word- sound correspondences. Consecutive training sets are depicted in consecutive charts (set 1: 5 sessions, set 2: 3 sessions, set 3: 4 sessions).

sessions in consecutive sets and performed significantly better in pointing to named letters at the end of the therapy but functional generalization was not shown since her health did not allow comprehensive evaluation of untrained items at the end of treatment. There was no generalization to untrained phonemes after the first set of trained phonemes in either participant. Moreover, there was no difference in the effectiveness of therapy between the two participants, indicating that individuals with PPA are as trainable as those with stroke. This is a very encouraging result for a degenerative disease such as PPA, as decline can be rapid and dramatic.

Unfortunately, we were not able to evaluate long-term effects of treatment in the PPA patient. Follow-up is often difficult in treatment studies of patients with neurodegenerative diseases. Other patients with logopenic variant PPA who had treatment of spelling and naming [3,13] had serious cognitive decline that did not allow follow-up investigations of therapy gains. Nevertheless, there is one therapy study of a logopenic PPA patient who received a naming/word retrieval intervention and showed generalization as well as retained therapy gains after 6 months [2]. Our stroke participant (WCR) retained the therapy gains at a 6-month follow-up, but during that time the PPA participant (FSE) had a major decline in her cognitive condition that did not allow further testing, indicating that 6-month follow-up may be a long time for some par-

Table 3
Follow-up evaluation of WCR on letter- and word-sound associations in all 3 trained sets after 6 months

| | letters | | | words | | |
|---|---|---|---|---|---|---|
| set | Baseline | End of therapy | Follow-up | Baseline | End of therapy | Follow-up |
| 1 | 0/10 | 9/10 | 8/10 | 4/10 | 9/10 | 8/10 |
| 2 | 5/10 | 8/10 | 6/10 | 6/10 | 8/10 | 7/10 |
| 3 | 5/10 | 7/10 | 8/10 | 7/10 | 9/10 | 7/10 |

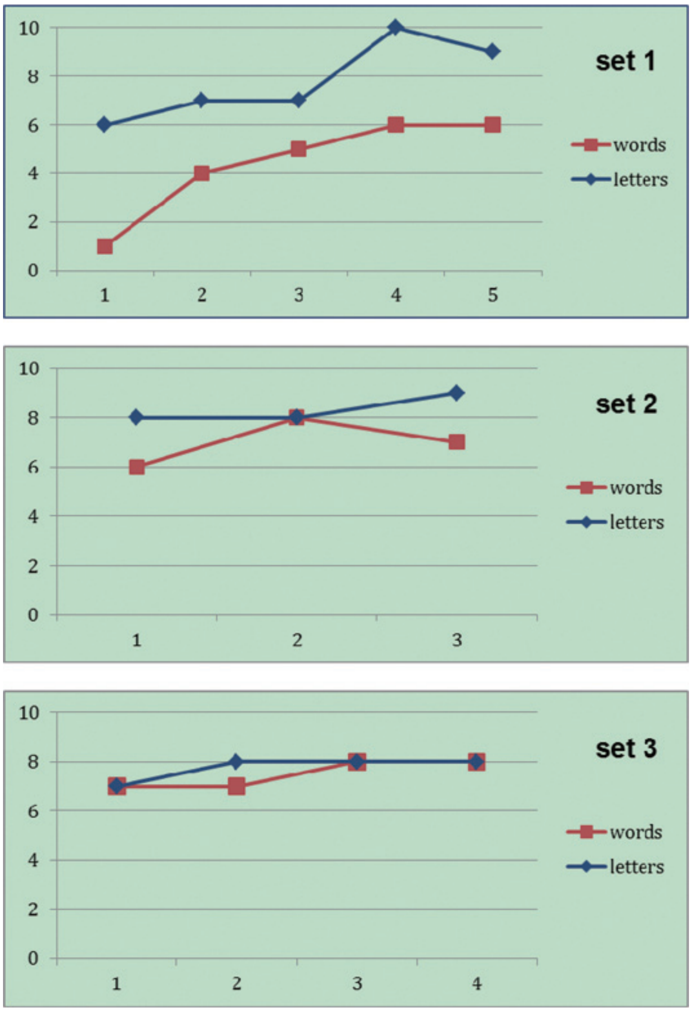

Fig. 3. Effect of training on trained items in each session for letter- and word-sound correspondences for patient WCR. Each dot corresponds to correct items per session for letter- and word- sound correspondences. Consecutive training sets are depicted in consecutive charts (set 1: 11 sessions, set 2: 9 sessions, set 3: 5 sessions).

ticipants with PPA. Therefore, future studies of PPA should consider shorter follow-up periods and perhaps repetition of the therapeutic procedures to improve retention.

The other difference between the two participants was that the one with stroke actually required more sessions to learn phoneme-grapheme associations than the one with PPA. This difference likely had more to do with differences in the underlying cognitive impairment in spelling than the neurological etiology of the dysgraphia. That is, when the PG mechanism is partially preserved (as in the case of the participant with PPA), training can occur in a shorter period of time. Overall, the response to treatment in PPA was good, despite the fact that she did not show generalization of effects after training the first set of phonemegrapheme and phoneme-word associations. However, even at that early stage, her trained phoneme-word associations showed a trend toward significant improvement, indicating that the therapeutic intervention improved other functions that were deficient, that is, word finding.

Even though WCR showed no statistically significant improvement in phoneme-word associations, examination of the graphs in Figs 2 and 3 show parallel improvement in phoneme-grapheme association and phoneme-word association indicating that he (like FSE and previously reported patients [27–29]) was also likely learning phoneme-grapheme associations via phoneme-word associations. These results indicate that the same therapeutic intervention may be equally effective for participants with different underlying deficits and etiologies. Spelling therapy of phoneme-to-grapheme conversion training with word-probes helped the participant with post-stroke aphasia strengthen and improve his phoneme-grapheme associations – which was a major weakness, along with a graphemic buffer deficit. The same therapy seemed to help the PPA patient to strengthen phoneme-grapheme conversion and improve access to orthographic representations – which were her major weaknesses. Improved word spelling with improvement in PGC can be expected on the basis of the 'summation hypothesis' [35] according to which lexical-semantic and sublexical mechanisms may interact in synergy and contribute together towards improvement of performance. In the only study that has looked at spelling patterns in different PPA variants, Sepelyak et al. [1] found that half of the logopenic variant PPA patients had a partially but not completely impaired sublexical mechanism (phoneme-to-grapheme conversion mechanism). Thus, strengthening this mechanism, particularly through phoneme-word associations may result in improved access to orthographic representations, perhaps via the frequent repetition of the words or the frequent practice of the cognitive processes involved in retrieving the spelling of taught words.

Improved word spelling by WCR despite his persistent graphemic buffer deficit may be explained by reliance on improved PGC to self-correct phonologically implausible errors in spelling. That is, even though he showed no generalization of training from trained phonemes to untrained phonemes in the first set when learning PGC, at the end of therapy, he clearly showed generalization of improvement in spelling to untrained tasks (word spelling to dictation) by applying PGC to untrained words. He maintained his ability to use PGC to compensate for his graphemic buffer deficit to spell short words six months later.

Although we were not able to follow the PPA participant long-term, it was encouraging that she showed improvement in spelling words despite having a condition in which language is generally declining. The long-term improvement in spelling by WCR, and his ability to apply the learned skill to an untrained task and untrained words, was also very encouraging.

## Acknowledgments

We are grateful to WCR and FSE for the generous donation of their time and effort to the completion of this project. We acknowledge support from NIH/NIDCD through R01 DC5375 and DC03681 for this work.
